# The Wisdom of the Moderns

**Published:** 1892-10-22

**Authors:** 


### The Hospital

An Institutional Journal of

**Science, Medicine, Nursing, and Philanthropy.**

*For Hospitals, Asylums, Infirmaries, Dispensaries, Medical Practitioners, Students, Nurses, and the Charitable Public.*

Vol. XIII.—No. 317.] [Oct. 22, 1892.

### The Wisdom of the Moderns.

"When pathologists were in the habit of looking only at the effects of disease—at lungs riddled with cavities, at the liver and kidneys shrunken and hardened by fibroid charge, at the brain torn by hæmorrhage—they might scoff at the efforts of the therapeutist to deal with such conditions. But these conditions have all had a starting point; and the patients who have ultimately died have lived through all the stages which have culminated in the ruin displayed on the *post mortem* table, while this same *post mortem* table has displayed scores of instances in which similar disease has been arrested at one or other of these stages. Processes are now the chief object of study and research; and in proportion as these are understood power will be gained of intervening and counteracting. As we learn how to set in motion morbid changes we learn how they may be arrested."

In terms like these Dr. Broadbent addressed the students of the Victoria University at the opening of the medical session at the beginning of the month. The address was like a dial plate held up to the shining sun of modern medicine. It showed to every comprehending man exactly where we are standing—exactly what time of day it is in the medical world. According to Dr. Broadbent our day has but well dawned; the sun is up, but it is the morning sun. We have long hours of daylight before us; and the task upon which we are set is the most arduous which has ever yet been given to man; the most arduous, but also the most beneficent and the most glorious.

Processes: that is the note of Dr. Broadbent's prophesy; it was also the note of Mr. Victor Horsley's address to the members of the Pathological Section of the British Medical Association the other day. Morbid anatomy, the *post mortem* table, shows us the last end of an operation, the ruin in its completion. We want to be at the beginning. The ruin is the very thing we wish not to see and study, but to prevent. But is it possible for us to be at the beginning? Can we actually and practically watch the first steps of diseased processes; and can we thoroughly understand them? Above all, can we lay a checking hand upon them and say, "Thus far hast thou gone, but no farther?" Or shall we be like Canute as he stood powerless on the sea shore, whither his courtiers had taken him to command back the inflowing tide? However we meet it, the crisis is upon us. The old, in medicine, has been to a large extent a scientific failure. What is the new to be?

An illustrative experiment may help our comprehension. Two rabbits and an experimenter are before us: beneath the skin of one rabbit a bacteria culture of a given kind and degree of virulence is injected. In due time inflammation appears at the point of injection, and perhaps also an abscess, but nothing more. The rabbit does not die, or show any sign of dying; on the contrary, it speedily becomes quite well. But now another rabbit is taken, and another bacteria culture. The second rabbit is as healthy and as strong as the former one, and the bacteria culture is of exactly the same kind, and of the same degree of virulence. The culture is injected; but at the very same moment a solution of chloral is also injected into a part of the rabbit's body far removed from the seat of the bacterial injection. What is the train of consequences we see now? A little inflammation? A little abscess? And then recovery? By no means! The result is rapid general infection of the rabbit's blood, lymph, and body, followed by inevitable death.

Here are facts of the profoundest significance. Let us understand them: still more, let us understand the lessons which they so convincingly teach. The unchloralised rabbit resisted the poison of the bacteria: the chloralised rabbit died. Why was this? Modern science, the science of the laboratory, makes the apparent mystery as clear as noonday, and as wonderful as clear. When the unchloralised rabbit was injected with the bacterial poison its normal forces of resistance were in perfect health and order. Those forces of resistance were its leucocytes, some at any rate of which acted the part of phagocytes. The phagocytes, warned apparently by some immediate effect of the injection upon the blood or tissues, gathered themselves together at the place of inoculation, and immediately joined battle with the bacteria. They engulphed them, as they engulph their ordinary food, and destroyed them. The result of the conflict was a little protective in-

ARMY MEDICAL LIBRARY WASHINGTON

flammation, followed perhaps in due course by a small abscess, and that was the end of the process. But the chloral, injected into the second rabbit at the time of the bacterial injection, paralysed the phagocytes; made them unable and unwilling to haste to the seat of battle, and to fight for the protecting citadel which was their own home and place of safety. Their citadel and themselves, as the result, were involved in a common ruin.

Is this sober fact? Or is it pathologist's fancy? It is sober fact. The leucocytes of the chloralised rabbit, when examined on the warm stage, showed their completely paralysed and helpless condition. Their movements were torpid; their pseudopodia were no longer pushed out from every part of their bodies as in the normal state; they were practically motionless and lifeless. The result, as we have said, was general infection and death.

The application of all this in medical practice and daily life is very obvious. The chloral, which destroys the resisting power of the phagocytes has its counterpart in a thousand circumstances of common experience. Let us take one of the most familiar. Alcohol, in considerable quantities, has precisely the same effect upon the phagocytes as chloral; it paralyses them; it makes them helpless in the presence of the enemy. As Dr. Broadbent graphically and eloquently puts it: "A single debauch may open the door to fever or erysipelas." Similarly a low and depressed state of the general health enfeebles and paralyses the phagocytes that resist bacterial disease, in the exact proportion that the body generally is enfeebled. So that, just as an exhausted and hunger-worn toiler at the end of a day could make no successful effort for his own life against a strong enemy, so the phagocytes of the same person could make little or no effort against an invading bacterial army. The poisonous atmospheres in which so many of us live furnish us with another example. But, indeed, the instances are innumerable in which men and women, voluntarily or involuntarily, live in circumstances which produce precisely the same effect upon them and their protecting phagocytes as the chloral produced upon the phagocytes of the rabbit, which the otherwise comparatively harmless inoculation so surely killed.

Now, in these experiments we have a complete history of a diseased process from beginning to end. The cause of the diseased process is known; its mode of operation is known; the continuous history is known; the effects are known; and the modifying circumstance of the injection of chloral—with its fatal results—are also known. But for the chloral the second rabbit would not have died. How often it happens in common experience that a modifying circumstance like the chloral, and not the disease itself, is the real thing that kills! We have the chloral of drink, the chloral of bad air, the chloral of neglected general health, the chloral of carelessly prepared or poisonous food, and a thousand others. As Dr. Broadbent urges—we must study much more profoundly the natural body and its environment, the chemistry of the natural body, its fluids, its solids, and their affinities for drugs, for food, and for poisons. All this must be done in the laboratory: and it must be done to some extent upon animals. There seems to be no help for it. There at any rate is the new and wonderful field of inquiry that lies before us. The knowledge of processes must be gained; that and the power to intervene at any stage of any particular process. Until this is gained, it would hardly be an exaggeration to say that nothing is gained. The old medicine has served us excellently for practical purposes; and it must continue to serve us until the new is conquered. But if ever fields were white unto the harvest, it is the fields of new knowledge and more beneficent power which lie before the medicine of the future.

